# Radiolysis as a solution for accelerated ageing studies of electrolytes in Lithium-ion batteries

**DOI:** 10.1038/ncomms7950

**Published:** 2015-04-24

**Authors:** Daniel Ortiz, Vincent Steinmetz, Delphine Durand, Solène Legand, Vincent Dauvois, Philippe Maître, Sophie Le Caër

**Affiliations:** 1 Institut Rayonnement Matière de Saclay, LIDyL et Service Interdisciplinaire sur les Systèmes Moléculaires et les Matériaux UMR 3299 CNRS/CEA SIS2M Laboratoire de Radiolyse, Bâtiment 546, F-91191 Gif-sur-Yvette, France; 2 Laboratoire de Chimie-Physique, UMR 8000 CNRS Université Paris Sud, Faculté des Sciences, Bâtiment 349, F-91405 Orsay, France; 3 CEA/Saclay, DEN/DANS/DPC/SECR/LRMO, F-91191 Gif-sur-Yvette, France

## Abstract

Diethyl carbonate and dimethyl carbonate are prototype examples of eco-friendly solvents used in lithium-ion batteries. Nevertheless, their degradation products affect both the battery performance and its safety. Therefore, it is of paramount importance to understand the reaction mechanisms involved in the ageing processes. Among those, redox processes are likely to play a critical role. Here we show that radiolysis is an ideal tool to generate the electrolytes degradation products. The major gases detected after irradiation (H_2_, CH_4_, C_2_H_6_, CO and CO_2_) are identified and quantified. Moreover, the chemical compounds formed in the liquid phase are characterized by different mass spectrometry techniques. Reaction mechanisms are then proposed. The detected products are consistent with those of the cycling of Li-based cells. This demonstrates that radiolysis is a versatile and very helpful tool to better understand the phenomena occurring in lithium-ion batteries.

## Abstract (web summary)

The degradation of organic solvents used in lithium-ion batteries reduces battery performance. Here, the authors present a radiolysis technique which is not only more efficient than conventional thermally activated ageing methods, but also allows mechanistic analysis of the degradation process.

## Graphical abstract

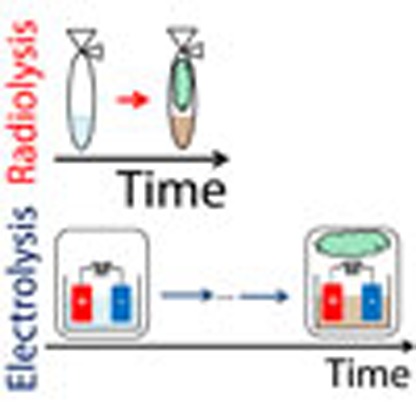

Lithium-ion batteries (LIBs) are ubiquitous in everyday life as they have very high gravimetric and volumetric energy densities^1^,^2^,^3^. To promote a higher energy storage by future generations^4^, major issues in battery developments must be faced and solved, not the least being the characterization of ageing processes^3^. Indeed, failure to do so adequately has led in the past to potentially disastrous safety issues^5^ or costly recalls. It is therefore paramount to have an in depth understanding of the underlying ageing phenomena^6^,^7^,^8^. Owing to the complexity of the whole LIB, many factors can induce safety problems but one key factor is the stability of the organic-solvent electrolyte^9^,^10^,^11^, which often cannot be studied by conventional thermally activated ageing methods. As a result, ageing studies can be lengthy, costly and usually remain purely qualitative^3^,^12^. Here, we demonstrate that radiolysis not only allows obtaining in a matter of hours what previously took months or even years to produce^13^,^14^,^15^,^16^,^17^, but also that it allows the quantitative and mechanistic study of these processes. Radiolysis has the potential of significant cost and time savings in the development of new battery electrolytes^18^.

This approach entails particularly two important benefits: the time needed to degrade the solvent is shortened (minutes–hours) as compared with the charge/discharge experiments (weeks–months). Second, processes at both short (ps–μs) and long-time scales (seconds–minutes) might also be studied, offering thus an understanding on temporal scales varying over some 12 orders of magnitude.

Commercial electrolytes are normally based on lithium salts in solutions of both linear alkyl carbonates and cyclic alkyl carbonates (Fig. 1). Indeed, the required properties of the electrolyte, such as conductivity or viscosity, can be optimized by combining solvents of different natures^19^. This paper focuses on the study of the stable degradation products formed at long times (seconds–minutes) in diethylcarbonate (DEC) and dimethylcarbonate (DMC) solutions (Fig. 1). Both give similar results; here we will present in detail those on DEC. DMC results are given in Supplementary Figs 1,2 and Supplementary Tables 1,2 and 3.

Gas phase products are identified using a gas chromatography-electron impact-mass spectrometry instrument (GC-EI/MS). A refined quantitative analysis is then performed using both an EI-magnetic sector mass spectrometer (EI/MS) and a Micro-Gas Chromatography (μ-GC) system. Liquid phase is also analysed by GC-EI/MS. Moreover, to further characterize the more complex liquid mixture, electrospray ionization-mass spectrometry (ESI/MS) technique is also employed. As radiolysis implies isomerization and the formation of numerous products, a combination of ESI, Differential Ion Mobility Spectrometry (DIMS) complements usefully the aforementioned mass spectrometry experiments^20^,^21^,^22^. Gas phase Infrared Multiphoton Dissociation (IRMPD) of a selected set of DIMS- and mass-selected ions is used to derive structural information^23^,^24^,^25^,^26^. To make the reading easier, a list of abbreviations is available in Supplementary Note 1.

All these complementary techniques enable us to characterize in detail the effect of radiolysis on DEC (and DMC) and to decipher the underlying reaction mechanisms. They are discussed and compared with reaction mechanisms occurring in electrolysis processes. In both cases, the same types of species are produced. Moreover, the detected products are consistent with the ones reported in the literature for the cycling of Li-based cells. This proves that radiolysis is a very useful tool to understand ageing phenomena in LIB.

## Results

### Gas phase results

**Figure 2:**
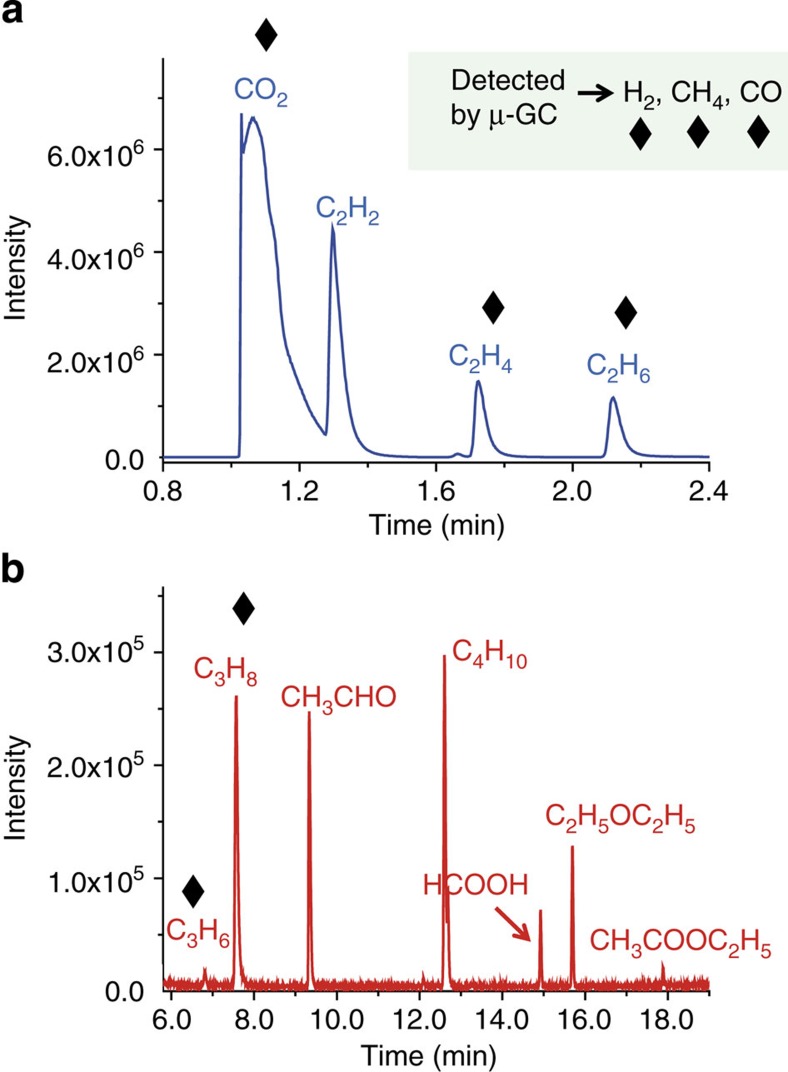
Gas decomposition products of DEC measured by GC-EI/MS after a 20-kGy irradiation. Gy stands for ‘Gray' and corresponds to 1 J kg^−1^. Except DEC itself, the part in blue (**a**) corresponds to the most intense peaks between 0.8 and 2.4 min, and the one in red (**b**) to smaller signals between 6 and 18 min. The top right molecules (H_2_, CH_4_, CO) are identified by μ-GC experiments. The compounds labelled with a diamond are identified in refs 15, 17.

Gas decomposition products of irradiated DEC obtained from GC-EI/MS are presented in Fig. 2. Different types of molecules are produced under irradiation: alkanes, alkenes and alkynes (for example, C_2_H_6_, C_2_H_4_ and C_2_H_2_); oxygenated molecules (aldehyde; ether; carboxylic acid). The different retention times of the products formed upon irradiation are given in Supplementary Table 4. It is important to notice that H_2_, CH_4_ and CO are not detected in the split mode by GC-EI/MS (Fig. 2) but that they are formed and measured by EI/MS and μ-GC.

**Figure 3:**
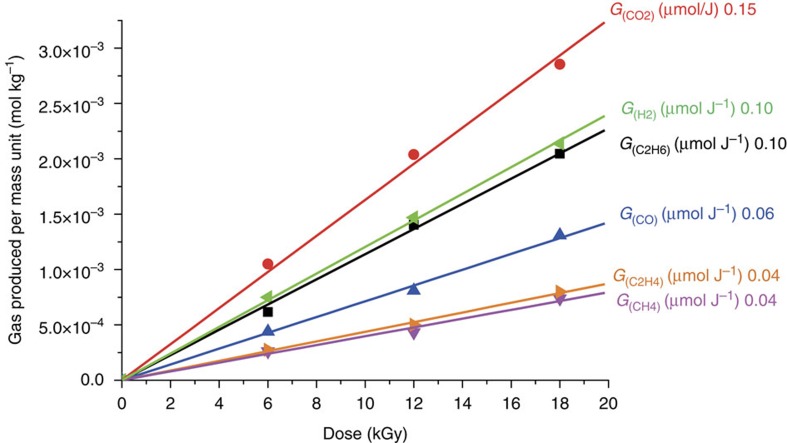
Evolution of the major DEC degradation products formed in the gas phase. They are obtained with the EI/magnetic sector mass spectrometer (EI/MS). For each gas, the radiolytic yield is given.

Having identified the molecules produced upon irradiation, the corresponding radiolytic yields *G*, which are defined as the amount of formed species per energy unit deposited in the sample, expressed in μmol J^−1^, can be measured. Main results are displayed both in Fig. 3 and Table 1. We checked that the yields measured with both irradiation setups were the same, within the uncertainty bars.

Main decomposition gases (Fig. 3) measured by EI/MS are CO_2_, H_2_, ethane and CO. In addition, other alkanes, such as CH_4_, propane and butane, and oxygenated products, such as acetaldehyde, diethyl ether or formic acid, are detected at lower quantities. Although ethyl acetate, acetylene and propene were detected, their yields were too low to quantify (estimated to be under 0.01 μmol J^−1^). Knowing the uncertainty bars estimated for EI/MS and gas chromatography (Table 1), the radiolytic yields obtained for CO_2_, H_2_ and CO are consistent with each other. A discrepancy exists in the case of the methane radiolytic yield. Indeed, in EI/MS experiments, the most abundant fragment of CH_4_ is the resulting ion CH_3_^+^at *m/z* 15. This ion is common to almost all identified molecules, leading to a greater uncertainty in this case.

### Liquid phase results

**Figure 4:**
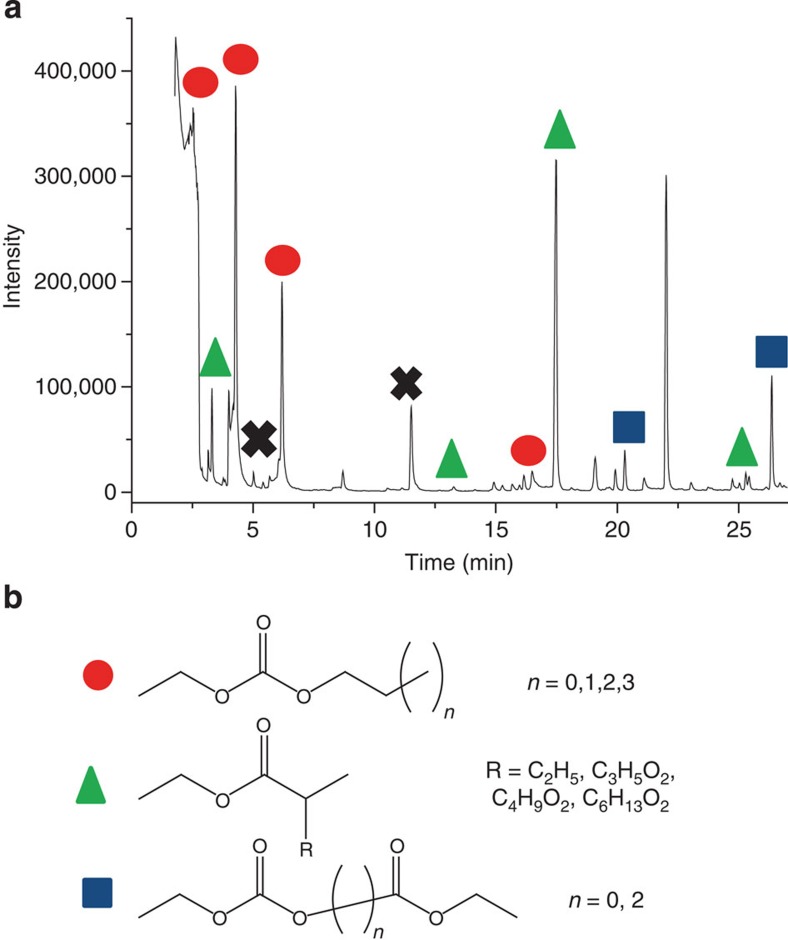
Liquid decomposition products of DEC measured by GC-EI/MS after a 100-kGy irradiation. (**a**) The GC-EI/MS chromatogram of the irradiated DEC. (**b**) Different types of degradation products represented with various colours and symbols. Black crosses represent other identified molecules, which do not belong to the above-mentioned types of degradation products.

Having identified and quantified the compounds formed in the gas phase, the evolution of the liquid phase is then studied in detail. The GC-EI/MS chromatogram obtained for irradiated DEC exhibits numerous peaks between 3 and 30 min (Fig. 4). Among the identified products, three types of degradation products are evidenced (Fig. 4). A detailed list of the identified products is given in Supplementary Table 5. The first one (symbolized by red circles) corresponds to a linear lengthening of the alkyl carbon chain of DEC. Different alkyl chain lengths ranging from *n*=1 (*t*_r_=4.6 min) to *n*=3 (*t*_r_=15.7 min) are detected. Blue squares symbolize compounds in which a C_2_H_5_-O-CO-O-***C***_***n***_***H***_***2n***_-CO-C_2_H_5_ bond is formed (with *n* equal to zero (*t*_r_=20.4 min) or two (*t*_r_=26.5 min)). Green triangles represent products for which a C-O bond cleavage and a branching in the alkyl chain occur. Finally, the C_2_H_5_-O-CH_2_CH_2_-OC_2_H_5_ (*t*_r_=5.6 min) and C_2_H_5_-O-CO-O-***CH***_***2***_***CH***_***2***_***-***O-C_2_H_5_ (*t*_r_=11.7 min) molecules, represented as black crosses, are also detected. Unfortunately, the remaining peaks which are not marked in Fig. 4 could not be identified with the NIST library. This led us to use an alternative ionization technique in order to better characterize the degradation products formed in the liquid phase.

A high-resolution ESI/MS spectrum obtained using a Fourier Transform Ion Cyclotron Resonance mass spectrometer (FT-ICR, 7T) for irradiated DEC was recorded (Supplementary Discussion and Supplementary Figs 3–5). Most of the detected peaks are consistent with the compounds obtained previously (Fig. 4 and Supplementary Table 5) but new signals also appear. Among them, the most abundant is the *m/z*=145.091 ion corresponding to the [***C***_***7***_***H***_***12***_***O***_***3***_***+H***]^***+***^ formula. In what follows, a special attention will be paid to the *m/z*=145.091 ion, hereafter denoted as 145.1, as it was not detected previously. The ion is generated in the gas phase and its structure is unraveled by IRMPD experiments.

### IRMPD experiments

**Figure 5:**
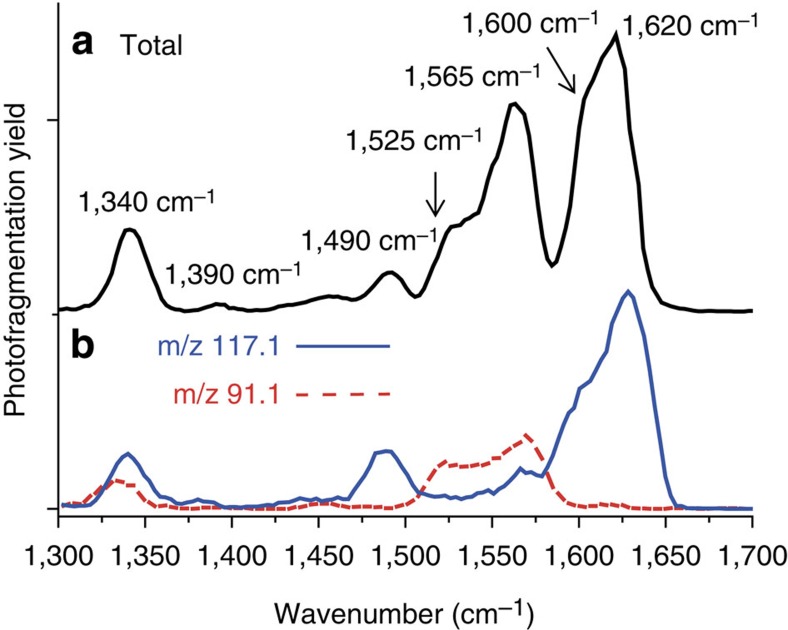
Total and mass-resolved IRMPD spectra from *m/z*=145.1 ion. **a** shows the experimental IRMPD spectrum (black straight line) of the *m/z*=145.1 ion in the 1,300–1,700 cm^−1^ range. Mass-resolved IRMPD spectra are shown in **b**. Two fragmentation channels are detected at *m/z*=117.1 (straight blue line) and at *m/z*=91.1 (dotted red line).

**Figure 6:**
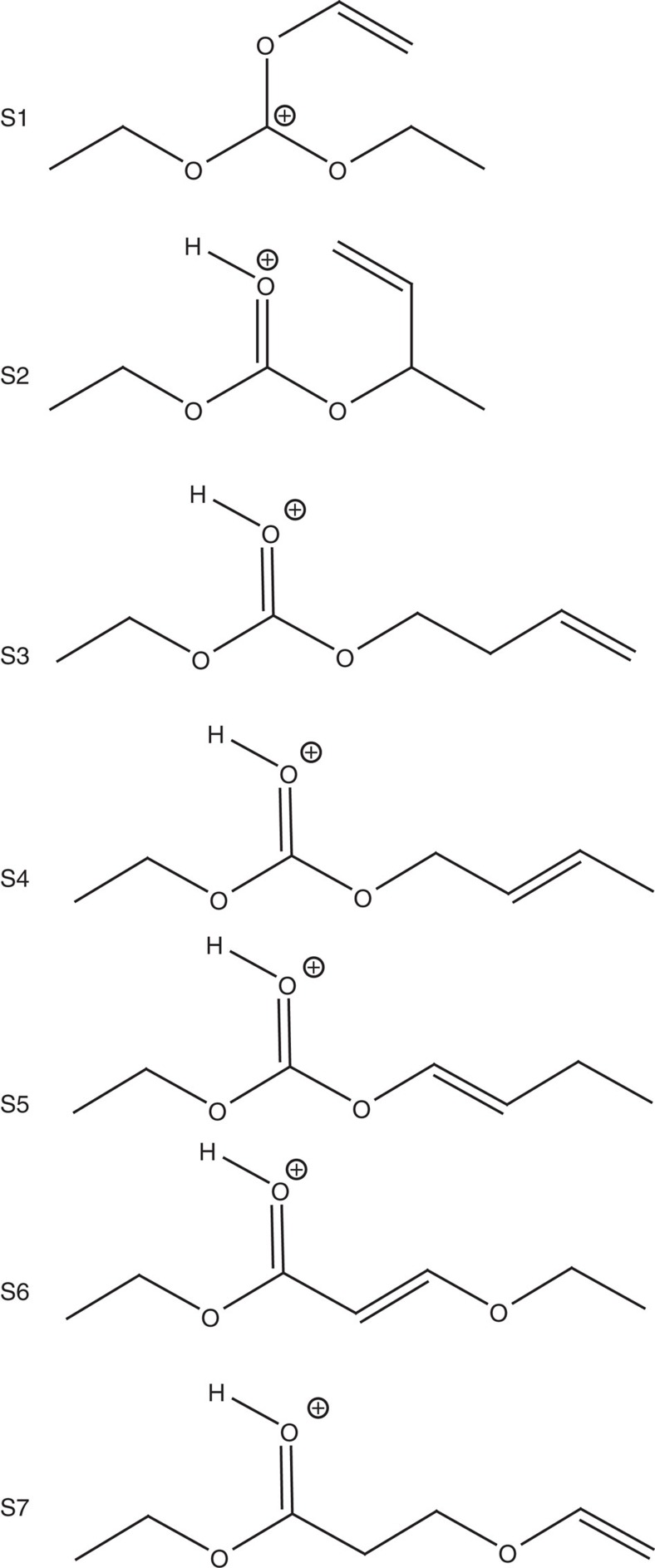
Considered compounds corresponding to the [*C*_*7*_*H*_*12*_*O*_*3*_*+H*]^*+*^ molecular formula. They were taken into account to elucidate the structure of the *m/z*=145.1 ion.

In these IRMPD experiments, two dissociation channels are observed at *m/z*=117.1 (loss of ethylene) and 91.1 (loss of C_4_H_6_; consistent with Collision Induced Dissociation experiments, see Supplementary Fig. 6). The IRMPD spectrum of the *m/z*=145.1 ion is dominated by two intense broad bands centred around 1,620 and 1,565 cm^−1^, respectively (Fig. 5). Two other weaker bands are observed at 1,490 and 1,340 cm^−1^, respectively. Moreover, DIMS experiments evidence that, at least, three isomers coexist at this *m/z* ratio (Supplementary Discussion and Supplementary Figs 7 and 8). Two main facts are consistent with this ion co-existence at *m/z*=145.1 ion: (i) the broad complex shape of the bands observed between 1,500 and 1,660 cm^−1^; (ii) the fact that each IRMPD fragment exhibits a specific wavelength dependence (chart *b* of Fig. 5 with the mass-resolved IRMPD spectra of *m/z*=117.1 and 91.1). Interestingly, the two bands located at 1,565 and 1,525 cm^−1^ are specific of the fragment ion *m/z*=91.1. To get a structural assignment of the different underlying species, Density functional theory (DFT)-computed infrared absorption spectra of different isomeric structures were compared with the experimental IRMPD spectra. All the seven structures (S_1_–S_7_) having the molecular formula [***C***_***7***_***H***_***12***_***O***_***3***_***+H***]^***+***^ and consistent with the expected chemical functions present in the ion and the radiolysis experiments were taken into account (Fig. 6). Depending on their infrared signature, three types of compounds were considered: (i) a carbocation (C_2_H_5_-O)_2_-***C***^***+***^-(OC_2_H_3_) (S_1_), (ii) protonated carbonates R-O-**COH**^**+**^-O-R' (S_2_–S_5_) and (iii) protonated esters R-O-**COH**^**+**^-R' (S_6_–S_7_). The calculated infrared spectra of the S_1_–S_7_ structures are reported in Supplementary Figs 9–12. Comparison of the specific bands centred at 1,565 and 1,525 cm^−1^, associated with the fragment ion *m/z*=91.1, matches nicely with the proposed carbocation structure (C_2_H_5_-O)_2_-***C***^***+***^-(OC_2_H_3_) (S_1_; Supplementary Fig. 10), which is the single structure dominated by two intense bands in this wavenumber range. The bands centred at 1,568 and 1,528 cm^−1^ are assigned to the O–C–O antisymmetric stretching and the C-O-CH=CH_2_ symmetric stretching bands, respectively. In the DFT-computed spectra of the protonated carbonate structures R-O-**COH**^**+**^-O-R' (S_2_–S_5_), shown in Supplementary Fig. 11, an efficient isomer differentiation remains complex. All theoretical infrared spectra are dominated by two intense signals calculated between 1,630–1,615 and 1,530–1,515 cm^−1^ and are attributed to the O–C–O antisymmetric stretch and carbonyl C=O symmetric stretching vibrational modes, respectively. As shown in Supplementary Fig. 11, the two computed vibrational modes are close in energy for all structures (S_2_–S_5_), which makes an efficient differentiation difficult. Furthermore, Supplementary Fig. 12 shows the theoretical spectra of protonated ester-type structures R-O-**COH**^**+**^-R' (S_6_–S_7_). Both computed spectra are dominated by the carbonyl C=O symmetrical stretching band, centred between 1,615–1,630 cm^−1^. Moreover, no structure S_1_–S_5_ could account for the experimental 1,490 cm^−1^ band. This band, assigned to the CH_3_ deformation mode, can be explained by the presence of the S_6_ structure (C_2_H_5_-O-**COH**^**+**^-C_2_H_2_-O-C_2_H_5_) whose calculated infrared spectrum predicts this vibrational mode at 1,493 cm^−1^. The last band, observed experimentally at 1,340 cm^−1^, corresponds to a CH bending vibrational mode. However, it does not give any further information as it matches to all calculated structures (S_1_–S_7_). These experiments enabled us to confirm structures associated to the *m/z*=145.1 ion. The combination of **S**_**1**_ (C_2_H_5_-O)_2_-***C***^***+***^-(OC_2_H_3_), of at least one carbonate structure (S_2_–S_5_) and of at least the ester structure **S**_**6**_ (C_2_H_5_-O-**COH**^**+**^-C_2_H_2_-O-C_2_H_5_) allows simulating a global infrared spectrum, which is consistent with the complex feature of the experimental spectrum recorded (Supplementary Fig. 13).

## Discussion

It is well known that the first effect of ionizing radiation in liquids is to excite and ionize molecules:


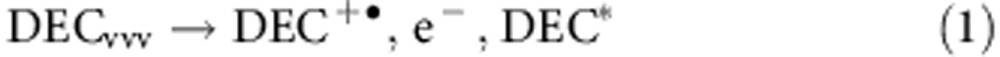


**Figure 7:**
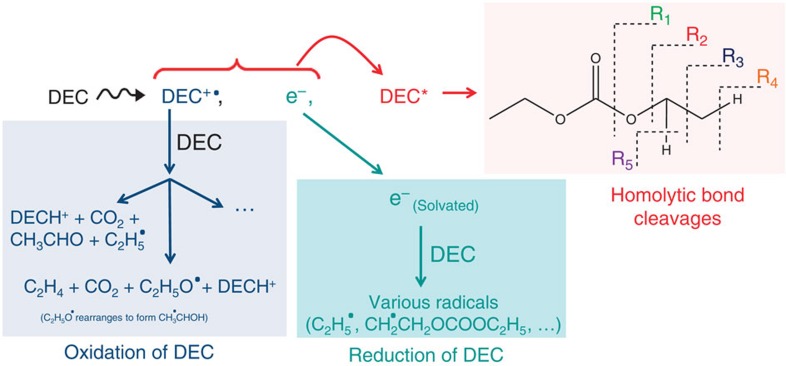
Different reactive channels of the radiolytic processes. The main channel, represented with the red colour, leads to the formation of the excited DEC molecule^27^, in which various homolytic bond cleavages (R1,…, R5) take place. The nature and amount of gases formed imply that the R2, R4 and R5 bond cleavages are the preferential ones. Among the possible reaction pathways, the reduction of DEC is indicated in the turquoise box^29^ and its oxidation is given in the blue box^28^. In battery applications, oxidation is observed at the positive electrode when the cell is overcharged at high voltages, and reduction takes place at the negative electrode.

The different role of these species is summarized in Fig. 7. First of all, it is important to remark that the static dielectric constant at room temperature of DEC is very low (2.8), meaning that the recombination of the electron with its parent radical cation DEC^+·^ is very fast, as evidenced by pulse radiolysis experiments performed at the picosecond timescale^27^. Moreover, this recombination of DEC^+·^ with the electron leads to the formation of the excited DEC molecule (Fig. 7). It was reported that the remaining radical cations DEC^+·^, which have not reacted with the electron, may induce a proton transfer reaction to another DEC molecule, leading to the formation of DECH^+^ and to the neutral species DEC(-H)^·^ (equation 2):^27^


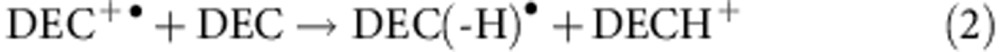


Other reaction channels between the radical cation and the DEC molecule are possible and can be proposed, similarly to the observations performed in the case of irradiated DMC^28^. In an electron spin resonance study (ESR)^28^, it was indeed shown that in DEC^+·^, an hydrogen-atom transfer takes place between the –CH_3_ group (and also between the adjacent –CH_2_- group) to the carbonyl oxygen, leading to the formation of CH_2_^·^CH_2_O(C=O^+^H)OC_2_H_5_ and of CH_3_CH^·^O(C=O^+^H)OC_2_H_5_, respectively. These species will then react with another DEC molecule (which act as a proton acceptor), and different bond cleavages can take place. Equations 3 and 4 can be proposed:


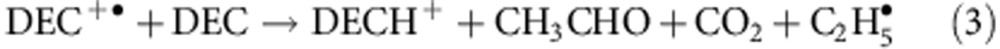



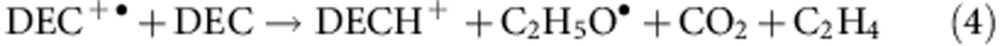


This leads to the formation of various radicals and molecules such as CO_2_. In equation 4, the C_2_H_5_O^·^ radical can rearrange to form the CH_3_C^·^HOH radical. Moreover, the formation of the ethyl radical (equation 3) can then lead, after H^·^ atom abstraction from the DEC molecule, to two radicals: CH_3_C^·^HOCOOC_2_H_5_ and ^·^CH_2_CH_2_OCOOC_2_H_5_ and to ethane formation. Equations 3 and 4 account for the formation of CH_3_CHO and of ethylene (Fig. 2) and are presented in the Fig. 7.

The remaining electrons, which have not reacted with the radical cation, will mainly solvate, leading to the formation of the 
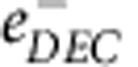
 species. Once they are solvated, and similar to reactions written in the case of DMC and ethylmethylcarbonate^29^,^30^, dissociative electron attachment can take place, leading to different bond cleavages and to the formation of various radicals such as C_2_H_5_^·^, which can then abstract an H^·^ atom from the DEC molecule (see above). This reductive path is shown in the Fig. 7. Nevertheless, pulse radiolysis experiments^27^ indicate that both reaction pathways, reductive and oxidative (Fig. 7), are minor under our experimental conditions. The various products reported in the present study are then mainly attributed to the reactivity of the DEC* molecule. Moreover, and similar to the observations performed in irradiated dodecane^31^, radiolysis can also form the excited radical cation, which can also lead to the excited DEC molecule. It is important to point out here that the same type of behaviour is expected in the case of DMC. These excited molecules can form directly small molecules such as CO, CO_2_ or H_2_…, and also lead to different homolytic bond cleavages (Fig. 7). For instance, bond cleavage R_1_ leads to the following radicals:


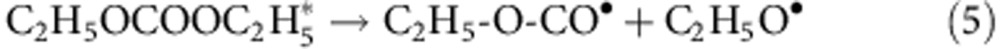


Similar equations can be written for R_2_–R_5_ (Fig. 7). Let us point out that radicals arising from C–H bond cleavage may also be secondary radicals formed after H^·^ atom abstraction from ethyl radicals, for example. The formation of the decomposition products arises then from different possible reaction mechanisms from the excited state: (i) direct production of small molecules such as CO, CO_2_ and H_2_…. from the excited state of DEC. The high CO_2_ yield measured, for example, cannot be explained by consecutive R_1_ and R_2_ bond cleavages, which are not very probable, but by a direct production from the excited state. The same explanation accounts for the high CO and CO_2_ yields measured in irradiated DMC (Supplementary Table 2). The difference in the relative proportions of gases formed from DMC and DEC implies that the reaction pathways from their respective excited state are different; (ii) radical recombination: the formation of diethylether can for instance arise from the recombination of radicals issued from R_1_ and R_2_. This recombination explains also the lengthening and branching of the alkyl chain of DEC (red circles and green triangles in Fig. 4); (iii) proton abstraction of radicals from the solvent (DEC) molecules, which are the most abundant ones. Therefore, this process is more probable than the previous one. For instance, the ethyl radical can abstract an hydrogen atom from the DEC molecule, leading to the formation of ethane and to the DEC(-H)^·^ radical. A similar reaction mechanism will explain the formation of H_2_ from H^·^ radicals.

The higher ethane yield measured as compared with methane (Table 1) implies then that R_2_ is preferred over R_3_ (Fig. 7). Ethylene can be formed by two different pathways: (i) an initial cleavage of C–H group via R5 (Fig. 7) could lead to an O–C bond cleavage, producing the CH^·^CH_3_ radical, which could rearrange to form ethylene and (ii) formation of the C_2_H_5_^·^ radical by pathway R2 followed by an H-atom abstraction. The nature and amount of gases measured from DEC imply that the preferential bond cleavages achieved are the C(=O)O–C and the C–H ones. The same trends are observed in DMC, and the C(=O)O–C bond cleavage leads to CH_3_^·^ radicals and then to methane formation. This is consistent with previous work, which has observed the CH_3_^·^ and CH_3_OCOOCH_2_^·^ radicals using ESR spectroscopy in irradiated frozen DMC^29^. Last, the high H_2_ yields measured here are in line with the dihydrogen yields determined for similar carbonyl compounds such as ketones^32^ and esters (*G*_H2_=0.099 μmol J^−1^ and *G*_H2_=0.079 μmol J^−1^, respectively)^33^,^34^.

Results obtained on the liquid phase evidences that the use of different analytical techniques is required for a detailed identification. Both ‘soft' and ‘hard' ionization techniques give consistent and complementary information. Although EI sorts out direct structural determination based on fragmentation patterns, ESI avoids fragmentation and generates intact degradation products. Moreover, ESI was important to unravel non-identified species in the chromatogram (Fig. 4), especially unsaturated ones. The ESI/IRMPD experiments have enabled to detect three main types of compounds: (i) a carbocation (S_1_), (ii) protonated carbonate R-O-**COH**^**+**^-O-R' and (iii) protonated ester R-O-**COH**^**+**^-R' (Fig. 6). For instance, the degradation product identified at *t*_r_=6.4 min (Fig. 4 and Supplementary Table 5) can convert into the S_3_–S_5_ structures (Fig. 6) after subsequent irradiation. However, the formation of the carbocation (S_1_) can only be explained by a 1,3-sigmatropic rearrangement process between S_1_ (C_2_H_5_-O)_2_-***C***^***+***^-(OC_2_H_3_) and S_2_ (C_2_H_5_-O-**COH**^**+**^-O-CH(CH=CH_2_)-CH_3_) structures, taking place within the mass spectrometer during the electrospray ionization. The S_1_ structure is then not due to radiation chemistry.

As above mentioned, our experimental data together with previous pulse radiolysis experiments^27^ indicate that the reactivity in the radiolysis experiments is mainly due to the excited state of diethylcarbonate (DEC*), leading then to various homolytic cleavages and to the formation of small molecules. Nevertheless, other reactive channels (implying the electron and the radical cation) exist and are summarized in Fig. 7. Let us point out that radiolysis leads to the formation of both the products arising from solvent reduction and from solvent oxidation.

In the field of batteries, the oxidative decomposition of the solvent is observed at the positive electrode when the battery is overcharged at high voltages, whereas reduction reactions of the solvent occur at the negative electrode. Even if numerous species take part in the complex surface chemistry on electrodes, the formation of the DEC^+·^ radical cation^35^ in overcharged cells and electron transfer at the negative electrode will lead to the formation of various radicals as described in both oxidative and reductive pathways of Fig. 7. The same intermediates (radicals) and the same molecules (CO_2_…) can then be found in the electrolysis experiments and in the radiolysis of DEC (Fig. 7). So, even if the molecules will not be formed in the same amounts in both techniques, radiolysis gives a precious insight into the processes taking place.

As mentioned before, one of the main goals of this study was to compare the species formed under irradiation with those formed by electrolysis. This comparison is depicted in Fig. 2, in which the gases detected in our study and in previous works on overcharged cells are labelled with a diamond^15^,^17^. Even if the solvents commonly studied in the field of LIBs are usually composed by mixtures of both linear and cyclic carbonates^36^, the major gases detected (H_2_, CO, CO_2_, CH_4_, C_*n*_H_2*n*+2_ or C_*n*_H_2*n*_ (refs 15, 17)) are the same, and the main oxidative and reductive gases (CO_2_, H_2_…) are measured and quantified in the present work. Our study evidences also the presence of minor oxygenated compounds (ester, ether…, Fig. 2). This can be linked to the possibility of radiation chemistry to deliver a significant amount of energy in a reasonable time, then enabling to evidence the formation of minor molecules, which is not easily accessible in batteries studies. In the liquid phase, Tarascon *et al*.^12^,^13^,^14^,^16^,^37^,^38^ have identified different sets of families in linear alkyl carbonates/EC-LiPF_6_ systems, also detected in our radiolysis experiments (Fig. 4 and Supplementary Fig. 14). These degradation products are based on: (i) (CH_2_-CH_2_-O)_*n*_ structure such as C_2_H_5_-O-C_2_H_4_-O-C_2_H_5_, (ii) (C_2_H_5_-O-CO-[O-C_2_H_4_]_*n*_-O-C_2_H_5_) as C_2_H_5_-O-CO-O-C_2_H_4_-O-C_2_H_5_ or (iii) (C_2_H_5_-O-CO-[O-C_2_H_4_]_*n*_-CO-O-C_2_H_5_), which are detected by GC-EI/MS (Supplementary Table 5). Last, it is possible, with the radiolysis tool, to study separately the reactivity of each solvent used in batteries, without or with LiPF_6_, and then to focus on different mixtures. Therefore, the role of each solvent and of the salt can be understood in details.

Radiolysis enabled us to generate stable degradation products of neat linear alkyl carbonates used in commercial LIBs. This entails three important advantages: (i) the time needed to degrade the electrolyte is shortened as compared with standard charge/discharge experiments, (ii) both short and long time-scale phenomena can be studied and (iii) the possibility to study the role of each solvent without/with salt. Our attention was first focused on both neat DEC and DMC. In the gas phase, H_2_, CO, CO_2_, alkanes (C_*n*_H_2*n*+2_)_*n*=1-4_, ethylene, acetylene and different kinds of oxygenated molecules were identified. In the liquid phase, more complex to analyse, different chain lengths and branching in the alkyl groups, as well as -(CH_2_-CH_2_-O)_*n*_ or (C_2_H_5_-O-CO-[O-C_2_H_4_]-O-C_2_H_5_) based structures have been found. The use of a different ionization technique (ESI as compared with EI) has also allowed identifying other decomposition products, especially unsaturated carbonates. The detected products are consistent with the ones reported in the literature for the cycling of Li-based cells.

Moreover, our experiments evidence the critical importance of the excited states of linear alkyl carbonates in radiolysis. These excited states can lead directly to the formation of small molecules (CO, CO_2_, H_2_…) in different proportions in DEC and in DMC, and to different bond cleavages forming various radicals. Once radicals are formed, radical recombination and proton abstraction from the solvent account for the detected compounds.

In conclusion, we show that the ‘radiolysis approach' used herein can provide a fast overview of the electrolyte decomposition phenomenon. Indeed, our results lead to similar sets of molecules as electrolysis. Radiolysis can then be very useful for a rapid screening of anti-ageing properties of new electrolytes.

## Methods

### Chemicals and sample preparation

Anhydrous grade DEC, DMC and potassium chunks (in mineral oil) were obtained from Sigma-Aldrich. To remove all water traces in the solvent, the solvent was pre-treated with potassium under argon atmosphere. The solution was then distillated under argon atmosphere in a flask containing a molecular sieve, which was before dried 24 h at 300 °C. The water amount was measured by a coulometric Karl–Fischer titrator and was never higher than 100 p.p.m. Before irradiation, the samples were degassed during 30 min by argon bubbling and placed in a Pyrex glass ampoule. They were then outgassed at approximately 3 mbar and subsequently filled with 1.5 bar of argon 6.0. This operation was repeated three times.

### Irradiation experiments

A Gammacell 3000 with a ^137^Cs source was used. The dose rate (5.1±0.2 Gy min^−1^, with 1 Gy=1 J kg^−1^) was determined using the Fricke dosimeter^39^. The total dose received by the sample was about 20 kGy, which is achieved here in 2–3 days.

Moreover, to get a significant amount of degradation products in the liquid phase, irradiations were also performed using the electron pulses of a Titan Beta, Inc. accelerator (10 MeV electrons with a pulse duration of 10 ns (ref. 40)), which delivers a higher dose to the sample than the Gammacell in a short time. We checked for these organic liquids that the degradation products obtained by these two irradiation setups are the same. A dose rate of 25 Gy per pulse was determined using the Fricke dosimeter^39^. To avoid a macroscopic heating of the sample during irradiation, the repetition rate was set to 2 Hz, for which the temperature of the sample remains below 40 °C as required in batteries. The 100-kGy dose is then delivered to the sample in roughly 30 min.

### Gas phase analytical methods

H_2_, CH_4_, CO and CO_2_ gases were quantified by gas chromatography (μ-GC-R3000, SRA instrument) using ultra-high purity helium as a carrier gas^41^. Moreover, to fully identify the degradation products formed in the gas phase, Gas Chromatography Mass Spectrometry (GC-MS) experiments were performed with an Agilent 6890 GC system interfaced with an Agilent 5973 MS equipped with an EI source, and a quadrupole mass analyser. The mass range is 4–160. Helium is used as the vector gas with a flow rate of 2 ml min^−1^. More details concerning the GC–MS apparatus are given in ref. 42. Finally, a gas mass spectrometer with a direct inlet equipped with an EI ionization source and a magnetic sector for mass analyser was used for a quantitative analysis (EI/MS). The mass range goes from 1 to 200 *amu* and the detection limit is about 1 p.p.m.

### Liquid phase analytical methods

GC-EI/MS experiments were carried out using a Waters GCT Premier-Time-of-Flight (TOF) mass spectrometer. Degradation products were separated with a (25 m × 0.25 mm) CP Sil 5 CB capillary column. The initial and final temperatures were 60 °C and 280 °C, respectively, with a temperature rate of 3 °C min^−1^. Helium was used as the vector gas with an inlet initial flow regulated at 1 ml min^−1^. The ion source was operated at 180 °C with an electron energy of 70 eV. EI spectra were obtained in the 10–800 *m/z* range.

For the electrospray-mass spectrometry (ESI-MS) experiments, the solutions were prepared by mixing 100 μl of alkyl carbonate, 1 ml of H_2_O/MeOH (40:60) and 2 μl of formic acid (98%). Solutions were infused with a syringe pump at a flow of 5 μl min^−1^ and a 5.5-kV voltage was applied to the capillary entrance. High-resolution mass spectra were obtained using a Fourier Transform Ion Cyclotron Resonance Spectrometer (Bruker FT-ICR, 7T). MS/MS Collision-Induced Dissociation spectra were also recorded for elucidating the structure of selected ions.

More direct structural information on mass-selected ions could also be derived from their IRMPD spectra recorded in the 1,200–1,800 cm^−1^ wavenumber range. These experiments were performed with a quadrupole ion trap mass spectrometer (Bruker Esquire 3000+) equipped with an electrospray ion source, which is coupled with IR lasers^23^. Tunable mid-infrared radiation produced by the free electron laser of CLIO (Centre Laser Infrarouge d'Orsay) was used^43^. The average laser power was on the order of 1,000 mW by setting the electron energy to 45 MeV. Upon resonant vibrational excitation, dissociation of the mass-selected ion is induced through the IRMPD mechanism. The infrared spectra are obtained by plotting the IRMPD efficiency as a function of wavenumber. Structural information can be obtained by comparing IRMPD spectra to the IR absorption spectra calculated for different isomeric structures of the ion.

### Computational details

DFT calculations were carried out with the Gaussian 03 package^44^. Geometry optimizations and the harmonic vibrational frequencies were computed by combining the B3LYP^45^,^46^ functional with the 6–311++G** basis set. The theoretical vibrational frequencies were then scaled by a factor of 0.98 (refs 47, 48). Each calculated band was convoluted assuming a Gaussian function having a full-width at half-maximum of 20 cm^−1^.

## Author contributions

D.O. and S.Le.C. conceived, designed and performed the experiments, analysed the results and wrote the paper. P.M. and V.S. helped in the IRMPD experiments and contributed to their interpretation. D.D., S.L. and V.D. helped during the GC–MS experiments.

## Additional information

**How to cite this article:** Ortiz, D. *et al*. Radiolysis as a solution for accelerated ageing studies of electrolytes in Lithium-ion batteries. *Nat. Commun*. 6:6950 doi: 10.1038/ncomms7950 (2015).

## Supplementary Material

Supplementary Information Supplementary Figures 1-14, Supplementary Tables 1-5, Supplementary Note 1, Supplementary Discussion and Supplementary References

## Figures and Tables

**Figure 1:**
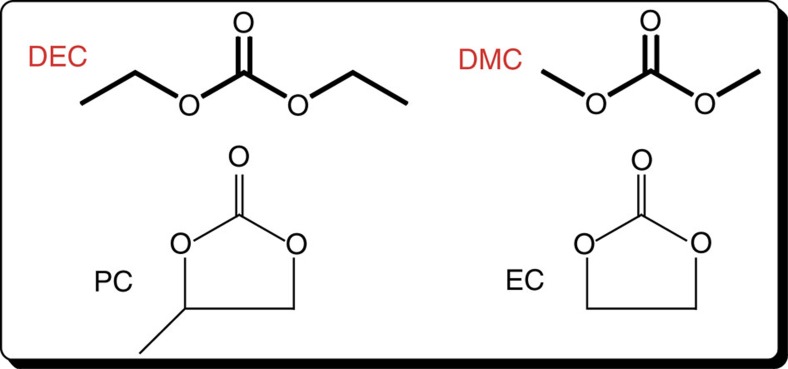
Common alkyl carbonates used in commercial electrolytes. Linear alkyl carbonates (diethylcarbonate, DEC, and dimethylcarbonate, DMC) and cyclic alkyl carbonates (propylene carbonate, PC, and ethylene carbonate, EC). DEC and DMC structures, written in bold, are studied here.

**Table 1:** Radiolytic yields of the degradation products formed in the gas phase.

	*G* (μmol J^−1^)μ-GC	*G* (μmol J^−1^)EI/MS
CO_2_	0.21	0.15
Ethane (C_2_H_6_)		0.10
H_2_	0.13	0.10
CO	0.05	0.06
Ethylene (C_2_H_4_)		0.04
Methane (CH_4_)	0.08	0.04
Formic acid (HCOOH)		0.03
Diethyl ether (C_2_H_5_OC_2_H_5_)		0.02
Acetaldehyde (CH_3_CHO)		0.02
Propane (C_3_H_8_)		0.01
Butane (C_4_H_10_)		0.01

GC, gas chromatography; EI/MS, electron impact-magnetic sector mass spectrometry.

They are expressed in μmol J^−1^ of gas produced after DEC irradiation and measured by both μ-GC (left) and EI/MS (right) techniques. The uncertainty bars are estimated to be 10% for the gas chromatography technique and 20% in the other case. The radiolytic yields can also be expressed in molecule per 100 eV by dividing the value obtained in μmol J^−1^ by the 1.036 10^−1^ factor.
