# Supplementary material for: Radiolysis as a solution for accelerated ageing studies of electrolytes in Lithium-ion batteries
Source: Nat Commun. 2015 Apr 24;6:6950. doi: 10.1038/ncomms7950 (PMC4421840; doi:10.1038/ncomms7950)
Supplement: Supplementary Information — Supplementary Figures 1-14, Supplementary Tables 1-5, Supplementary Note 1, Supplementary Discussion and Supplementary References [file ncomms7950-s1.pdf]

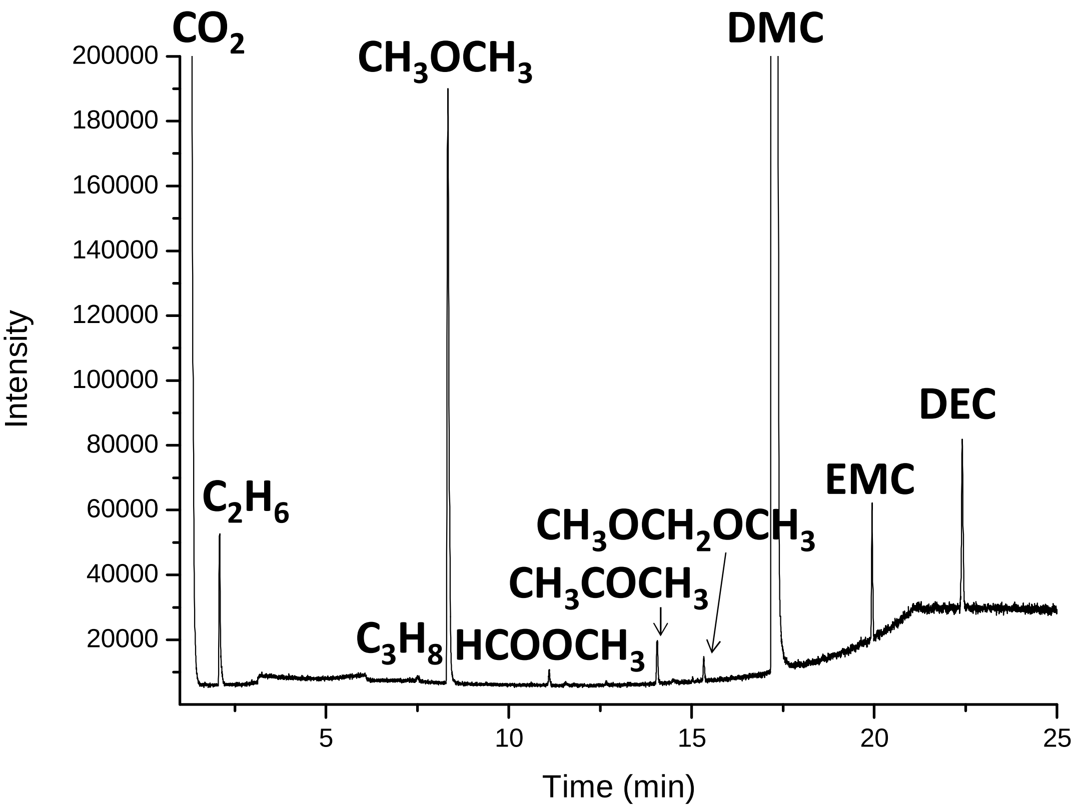

Supplementary Figure 1: Gas decomposition products of irradiated DMC. Decomposition products are measured by GC-EI/MS after a 20 kGy irradiation. EMC stands for ethylmethyl carbonate ($CH_3OCOOC_2H_5$).

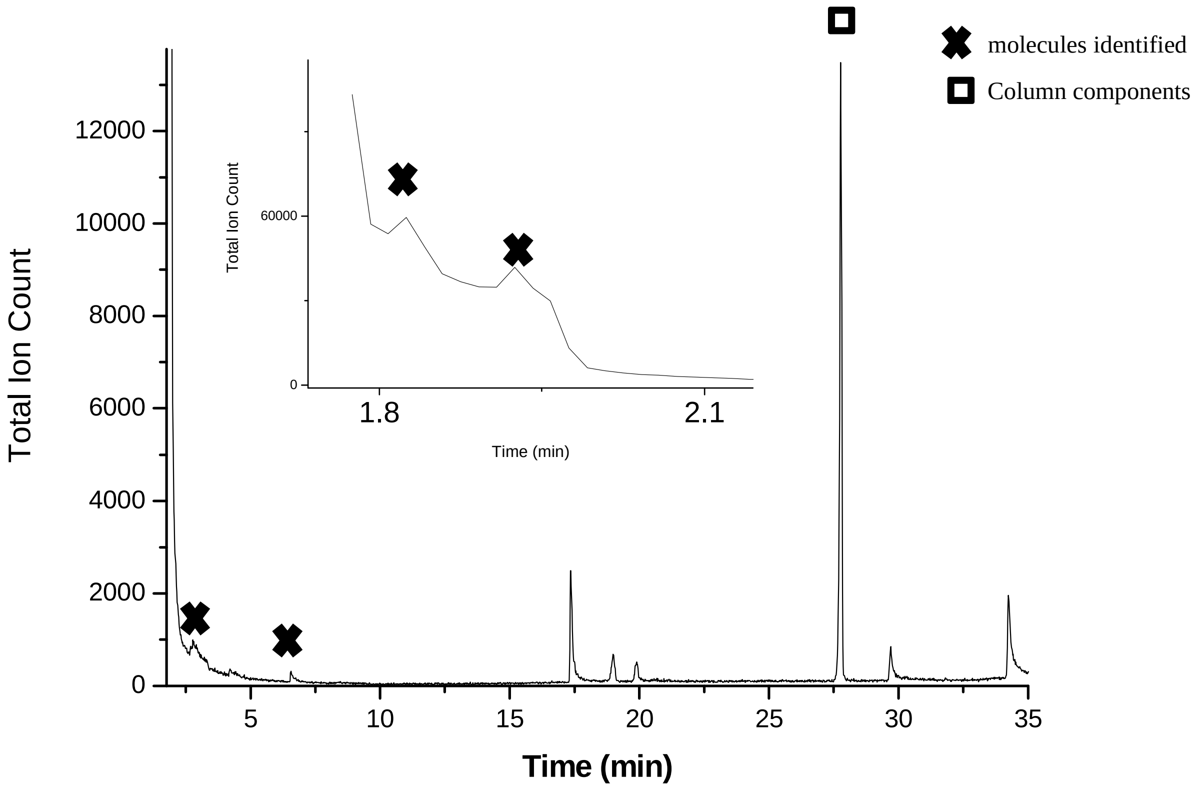

Supplementary Figure 2: Liquid decomposition products of irradiated DMC. The GC/EI-MS chromatogram was obtained for an irradiation at a dose of 100 kGy.

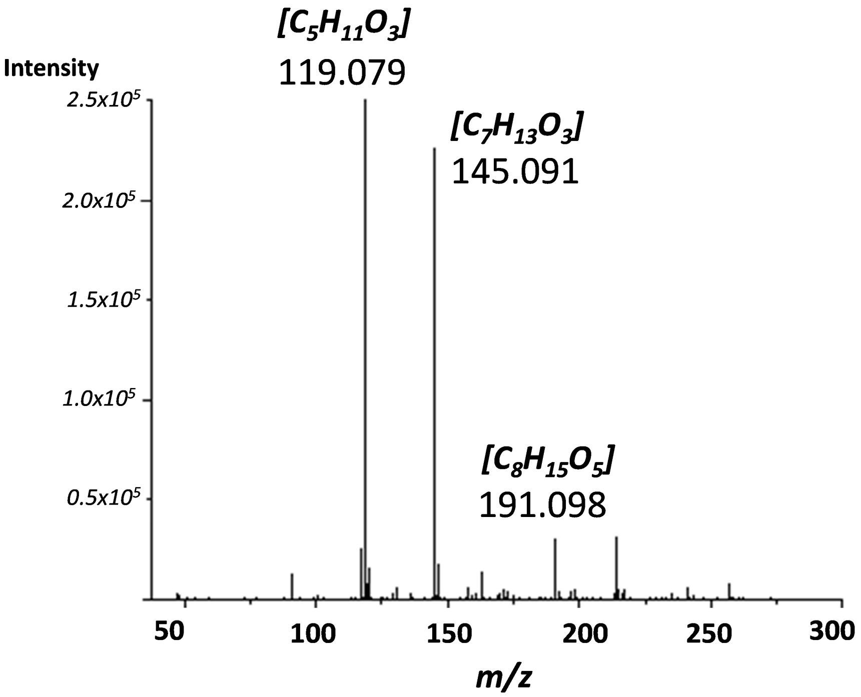

Supplementary Figure 3: ESI/MS spectrum of DEC irradiated at a dose of 100 kGy. The signal observed at *m/z* 214.093 corresponds to a pollutant present in the trap and has therefore not to be considered here. The solutions were prepared by mixing 100 µL of alkyl carbonate, 1 mL of H_2O/MeOH 40:60 and 2 µL of formic acid (98%).

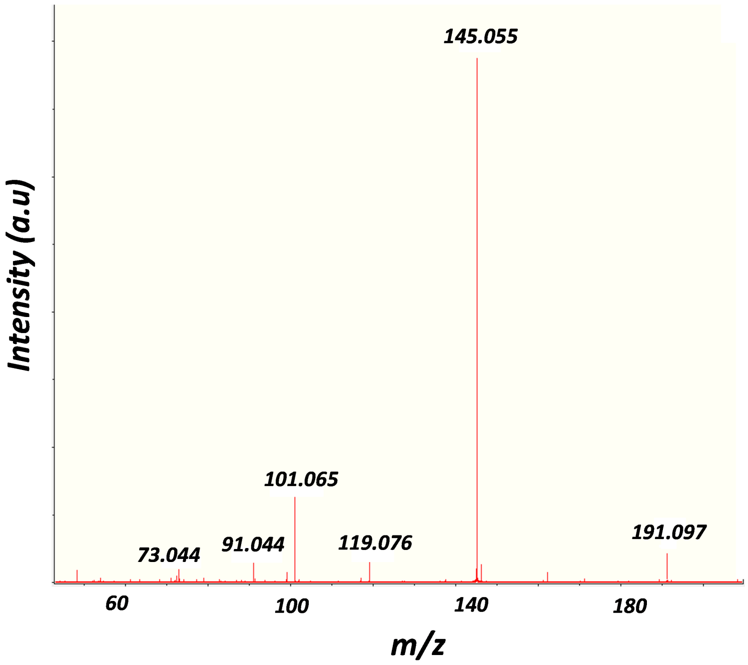

Supplementary Figure 4: MS/MS spectrum of the *m/z* 191.098 ion. The solutions were prepared by mixing 100 µL of alkyl carbonate, 1 mL of H_2O/MeOH 40:60 and 2 µL of formic acid (98%).

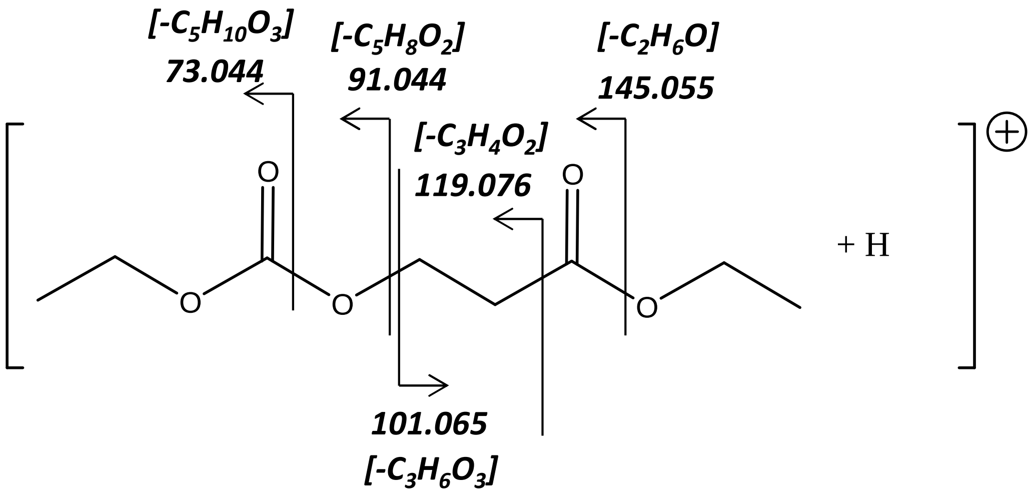

Supplementary Figure 5: Proposed MS/MS fragments for the $[C_8H_{14}O_5+H]^+$ ion (*m/z* 191.098). *m/z* values are recorded using the FT-ICR (7 T) mass spectrometer. Neutral loss structures are given in brackets.

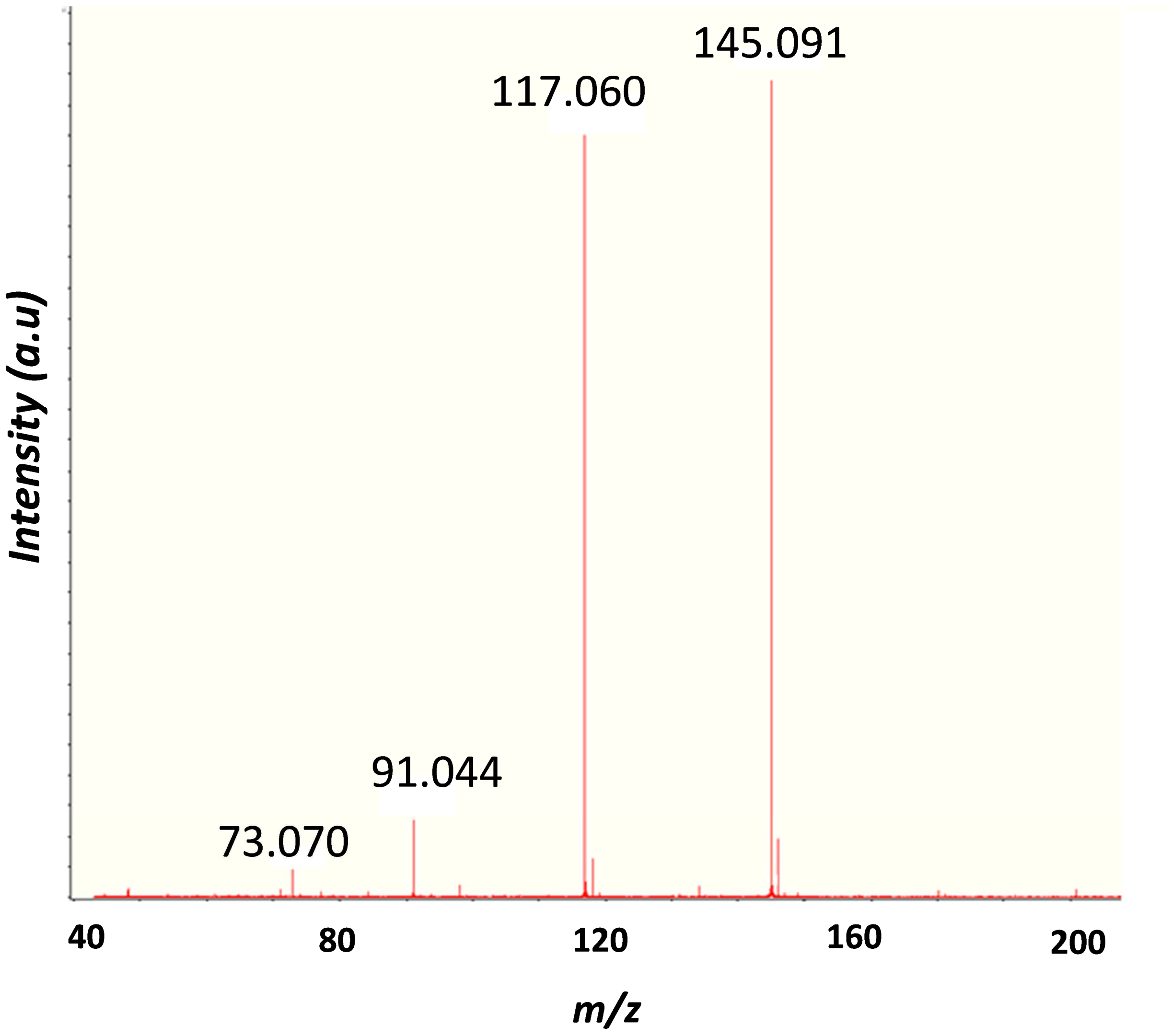

Supplementary Figure 6: MS/MS spectrum of the *m/z* 145.091 ion. It was recorded by using the FT-ICR mass spectrometer. The solutions were prepared by mixing 100 µL of alkyl carbonate, 1 mL of H_2O/MeOH 40:60 and 2 µL of formic acid (98%). The Collision Induced Dissociation MS/MS spectra of the fragment ion $[C_7H_{12}O_3+H]^+$ at *m/z* 145.091 are presented here. Two main dissociation channels are observed at *m/z* 117.060 and 91.044 which are consistent with losses of 28.031 (ethylene) and 54.046 (C_4H_6), respectively.

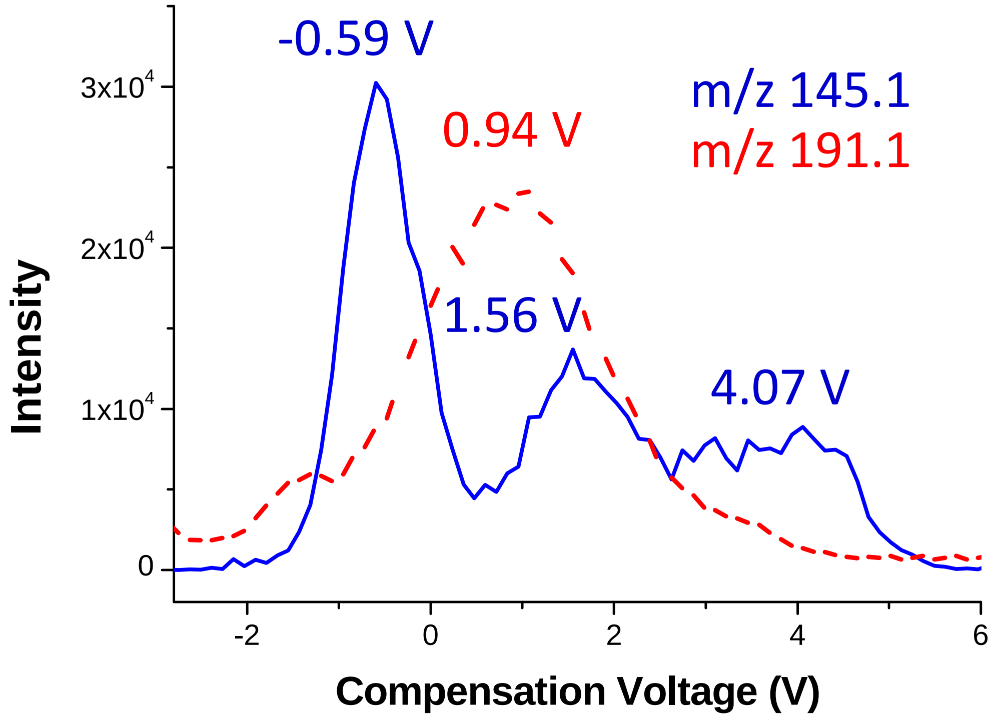

Supplementary Figure 7: DIMS chromatograms. Mobility spectra of *m/z* 145.1 (blue straight line) and 191.1 (red dotted line) isomers obtained in irradiated DEC as measured by ESI/DIMS/MS.

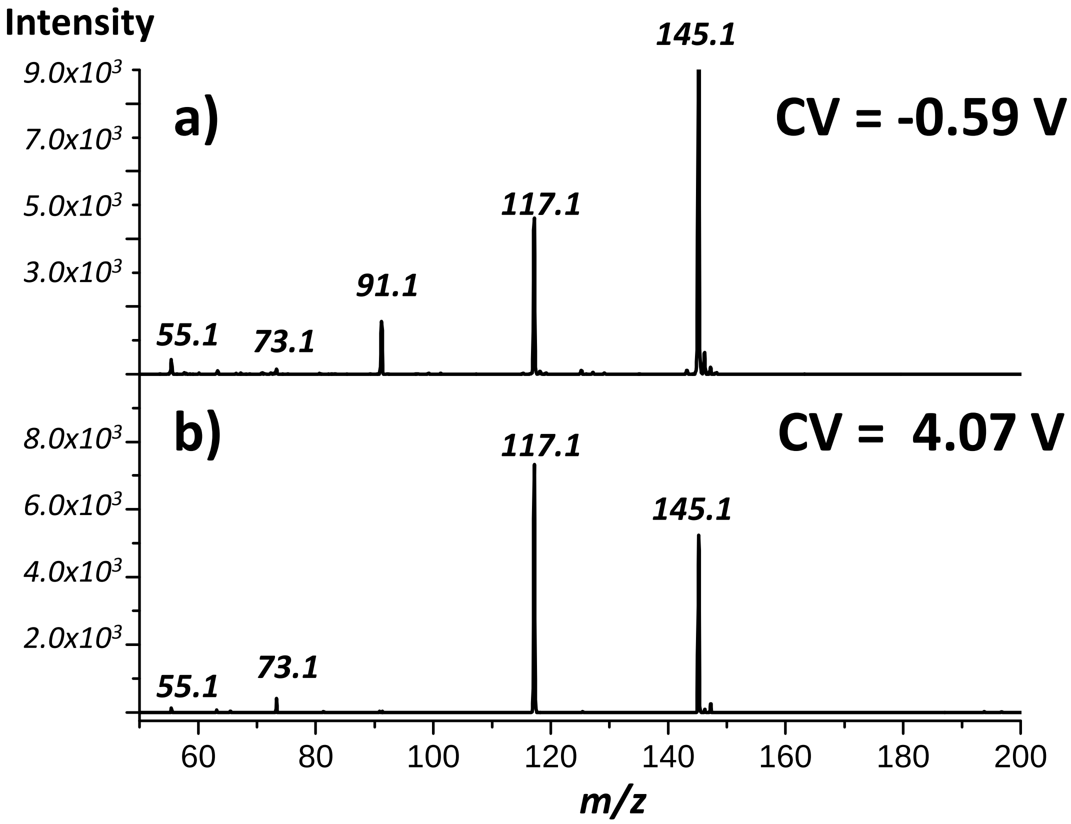

Supplementary Figure 8: MS/MS spectra comparison of the *m/z* 145.1 ion at different compensation voltages. CV is fixed at a) -0.59 V and b) 4.07 V before the MS/MS experiment.

S_1

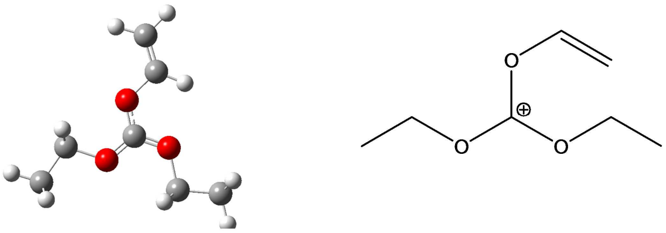

S_2

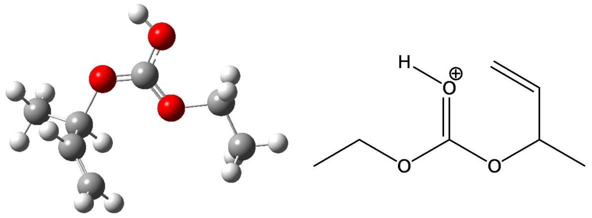

S_3

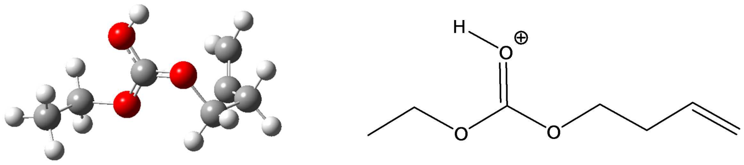

S_4

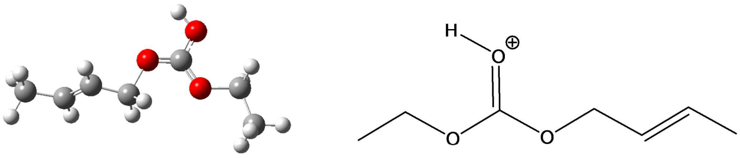

S_5

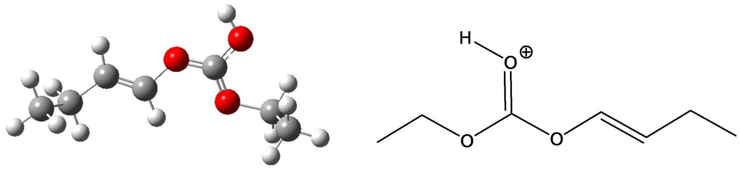

S_6

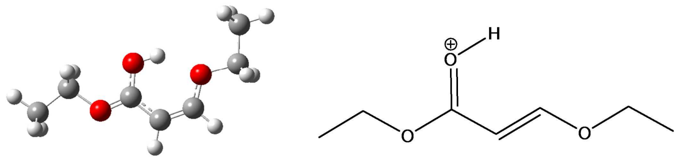

S_7

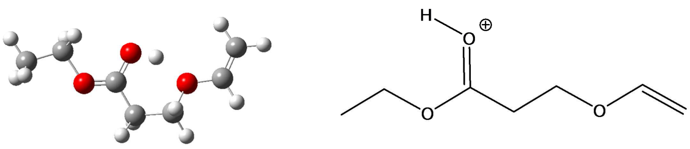

Supplementary Figure 9: Optimized structures for the considered compounds corresponding to the [$C_7H_{12}O_3$+H]$^+$ **molecular formula.** Calculations are carried out at the B3LYP/6-311++G** level of theory. All theoretical calculations are available upon author request.

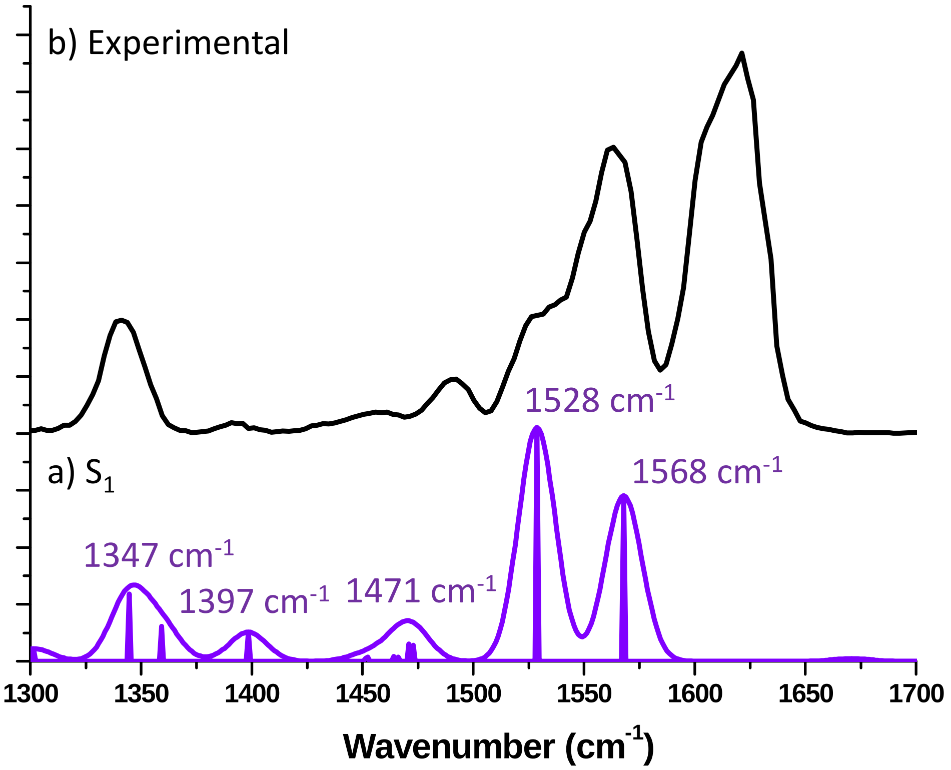

Supplementary Figure 10: Experimental IRMPD spectra of the *m/z* 145.1 ion with the calculated spectrum of the S1 structure. Chart a shows the calculated spectrum of the S1 structure (Supplementary Figure 9). Calculations (purple straight line) are carried out at the B3LYP/6-311++G** level of theory. All theoretical calculations are available upon author request.

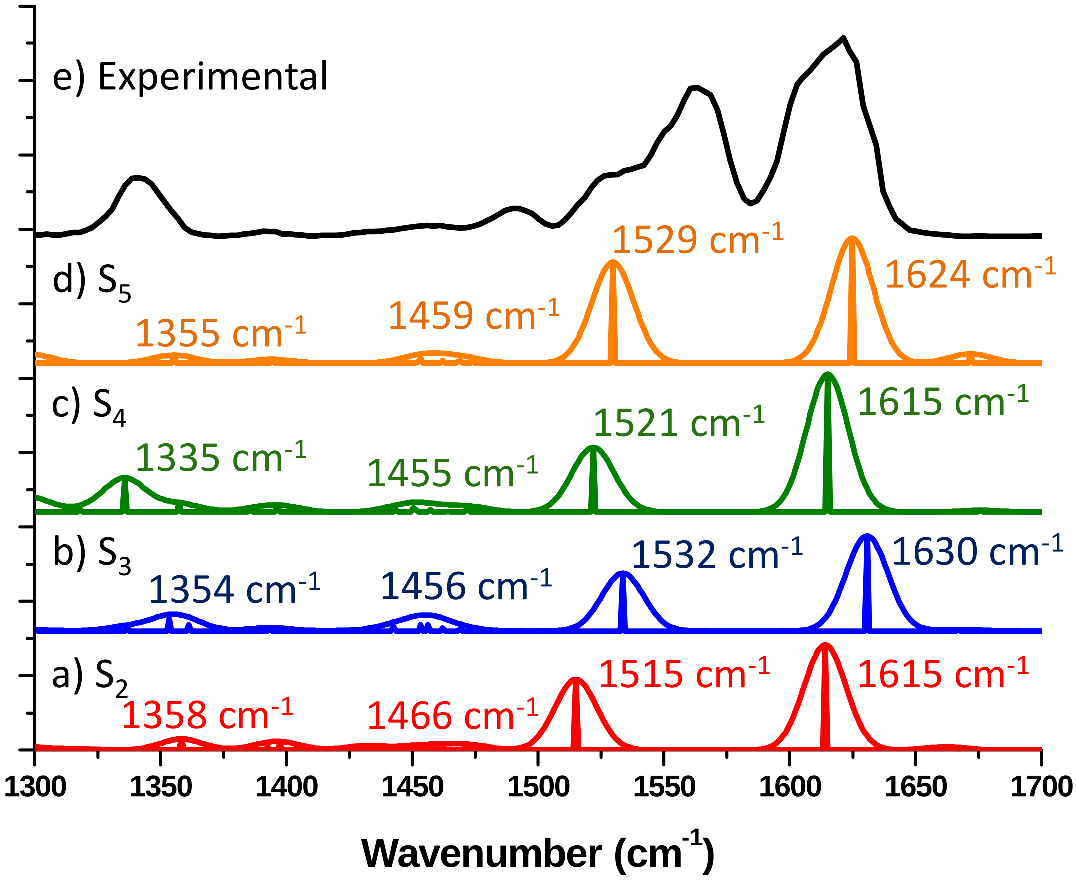

Supplementary Figure 11: Experimental IRMPD spectra of the *m/z* 145.1 ion with the calculated spectra of the S2-S5 structures. Charts a-d show the different calculated spectra of the S2-S5 structures (Supplementary Figure 9). Calculations are carried out at the B3LYP/6-311++G** level of theory. All theoretical calculations are available upon author request.

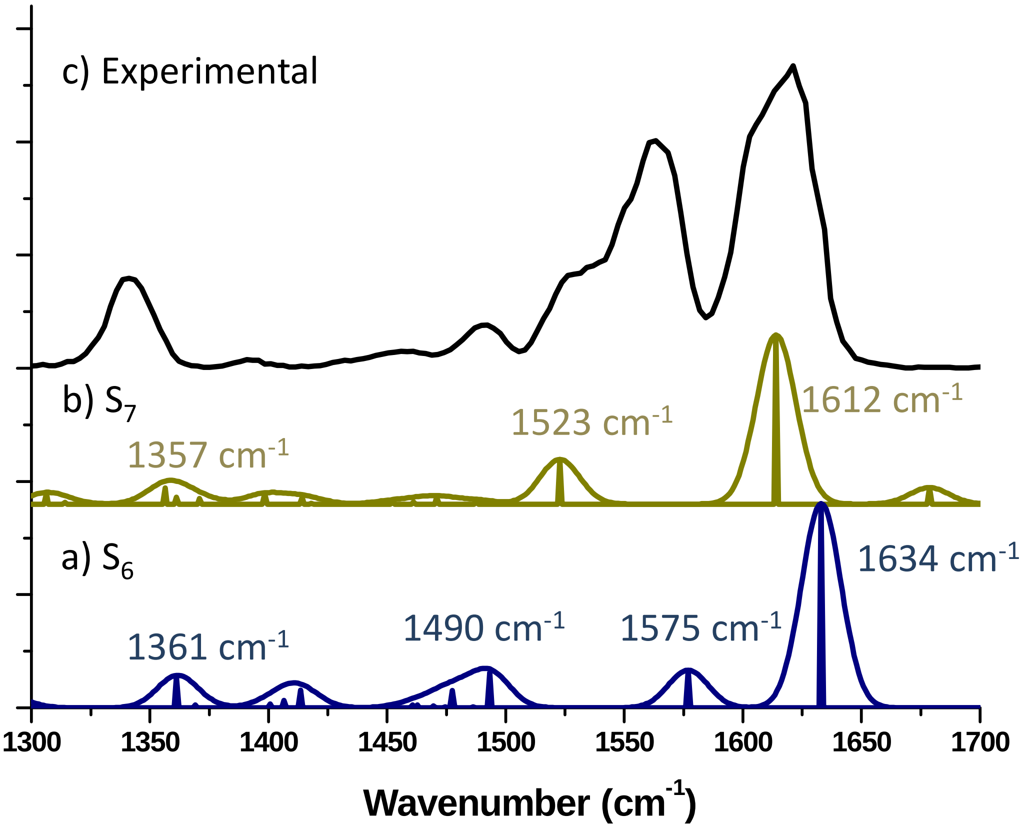

Supplementary Figure 12: Experimental IRMPD spectra of the *m/z* 145.1 ion with the calculated spectra of the S6-S7 structures. Charts a and b show the calculated spectra of the S6 and S7 structures, respectively (Supplementary Figure 9). Calculations are carried out at the B3LYP/6-311++G** level of theory. All theoretical calculations are available upon author request.

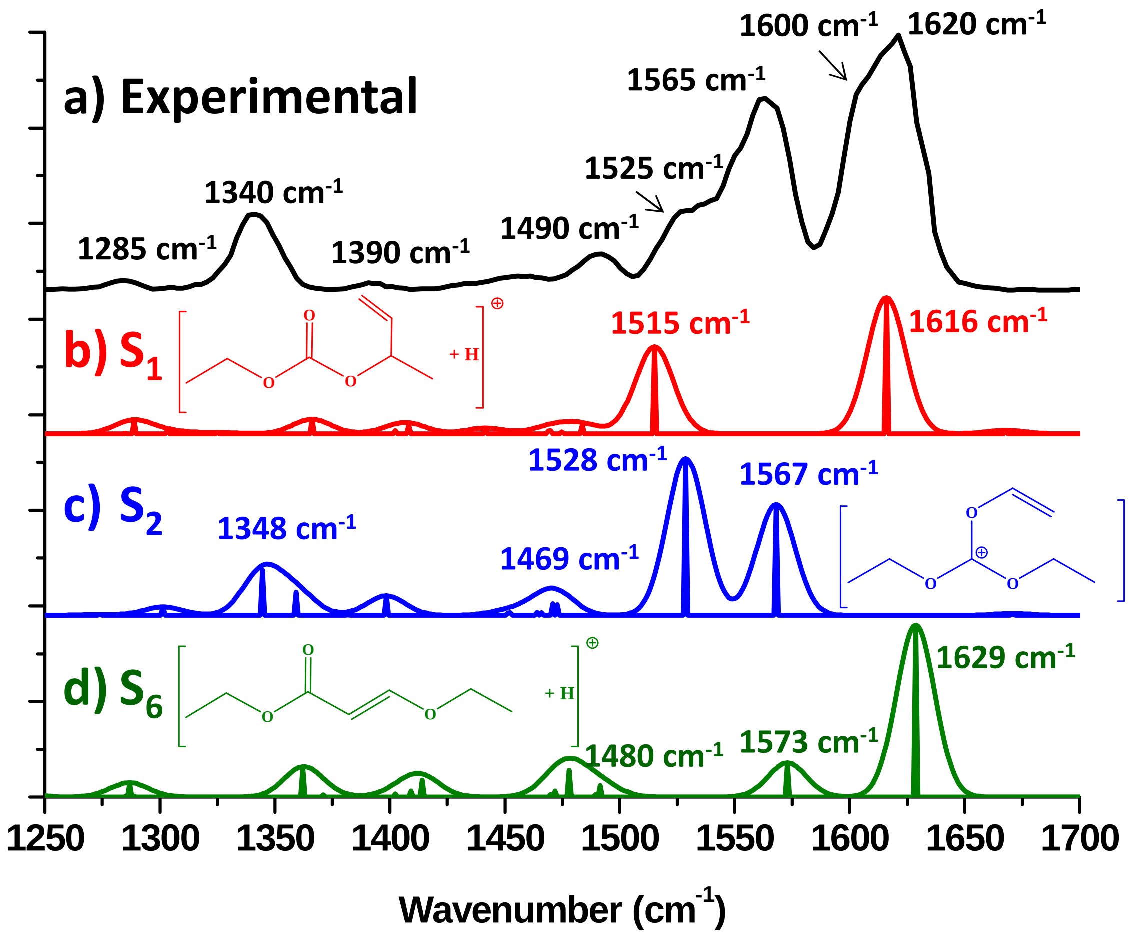

Supplementary Figure 13: Comparison of the Experimental IRMPD with S1, S2 and S6 theoretical infrared spectra. Chart a shows the experimental IRMPD spectra (black straight line) of the *m/z* 145.1 ion in the 1250-1700 cm^{-1} range. Theoretical IR spectra of S1 (red straight line), S2 (blue straight line) and S6 (green straight line) are represented in charts b, c and d respectively. All theoretical calculations are available upon author request.

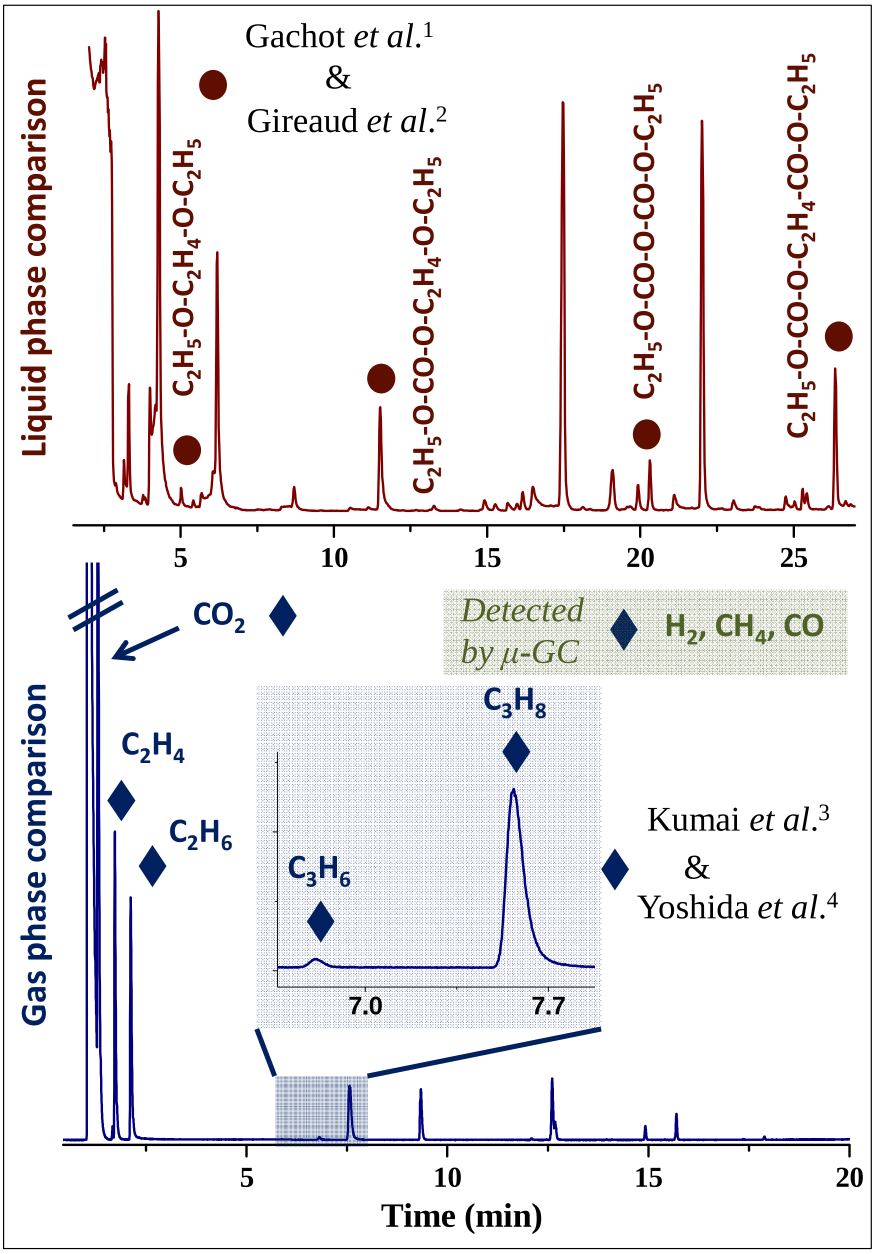

Supplementary Figure 14: Liquid and gas phase radiolysis/electrolysis comparison. Upper panel, GC-EI/MS chromatogram of the irradiated DEC at a dose of 100 kGy. The compounds labeled with a circle are identified in Supplementary References 1,2. Lower panel, gas decomposition products of DEC measured by GC-EI/MS after a 20 kGy irradiation. The top right molecules (H_2, CH_4, CO) are identified by μ-GC experiments. The compounds labeled with a diamond are identified in Supplementary References 3,4.

Molecule	t_r (min)	Molecule	t_r (min)
Argon	0.98	Acetone	14.05
Carbon Dioxide	1.17	Dimethoxymethane	15.32
Ethane	2.07	DMC	17.23
Propane	7.54	EMC	19.94
Dimethyl ether	8.33	DEC	22.41
Methyl formate	11.13		

Supplementary Table 1: GC/EI-MS retention times for irradiated DMC. The sample was irradiated at a total dose of 20 kGy. The most intense peaks correspond to Ar, CO_2, dimethyl ether and DMC at retention times equal to 0.98, 1.17, 8.33 and 17.23 min respectively. EMC stands for ethylmethyl carbonate ($CH_3OCOOC_2H_5$).

	G (µmol J^{-1}) GC	G (µmol J^{-1}) EI/MS
CO	0.19	0.24
CO_2	0.18	0.18
Methane (CH_4)	0.11	0.16
EMC		0.12
H_2	0.10	0.09
DEC		0.07
Dimethyl ether (CH_3OCH_3)		0.06
Ethane (C_2H_6)		0.02
Acetaldehyde (CH_3 CHO)		0.02
Methyl formate		0.02

Supplementary Table 2: Radiolytic yields for products formed in the gas phase for irradiated DMC. They are expressed in µmol J^{-1} of gas produced after DMC irradiation by both gas chromatography (left) and EI magnetic sector mass spectrometry (right) techniques. The error bars are estimated to be 10% for the gas chromatography technique and 20% in the other case. EMC stands for ethylmethyl carbonate ($CH_3OCOOC_2H_5$).

Retention time (min)	EI ions Intensity >10%	Proposed structure	Molecular formula	*m/z*
1.8	14, 15, 29, 30, 30, 44, 45, 59	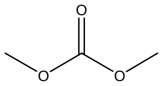	$C_3H_6O_3$	90.032
1.9	15, 27, 28, 29, 30, 45, 59, 77	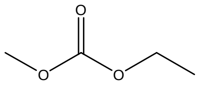	$C_4H_8O_3$	104.047
2.6	15, 29, 45, 59	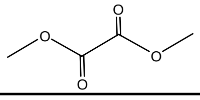	$C_4H_6O_4$	118.026
6.1	59, 74, 103	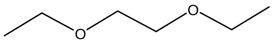	$C_6H_{14}O_2$	118.099

Supplementary Table 3: List of products formed in the liquid phase for irradiated DMC. The sample was measured by GC-EI/MS and irradiated at a dose of 100kGy. Due to the small size of the DMC molecule, only few peaks are observed in the chromatogram. The identified peaks of the DMC degradation products are CH_3-O-CO-O-***C_2H_5*** (t_r = 1.9 min), CH_3-O-CO-CO-O-CH_3 (t_r = 2.6 min) and C_2H_5-O-C_2H_4-O-C_2H_5 (t_r = 6.1 min).

Molecule	t_r (min)	Molecule	t_r (min)
Argon	0.98	Acetaldehyde	9.32
Carbon Dioxide	1.06	Butane	12.60
Acetylene	1.31	Formic acid	14.93
Ethylene	1.72	Diethyl ether	15.71
Ethane	2.11	Ethyl acetate	17.88
Propene	6.83	DEC	22.40
Propane	7.57		

Supplementary Table 4: GC/EI-MS retention times for the degradation products in the gas phase for irradiated DEC. The sample was irradiated at a total dose of 20 kGy. The most intense peaks correspond to Ar, CO_2 and DEC at retention times equal to 0.98, 1.06 and 22.40 min respectively.

Retention time (min)	EI ions Intensity >10%	Proposed structure	Molecular formula	*m/z*
3.0	45, 59, 63, 73, 91,117	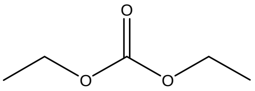	$C_5H_{10}O_3$	118.062
3.3	41, 57, 74, 85, 102	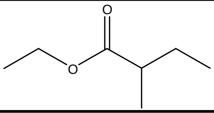	$C_7H_{14}O_2$	130.099
4.6	41, 59, 63, 90, 91, 105	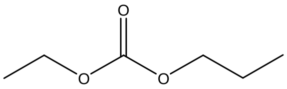	$C_6H_{12}O_3$	132.078
5.6	59, 74, 103	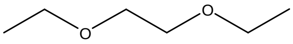	$C_6H_{14}O_2$	118.099
6.4	41, 45, 55, 56 57, 63, 73, 91	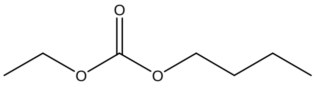	$C_7H_{14}O_3$	146.094
11.7	118, 91, 89, 72, 59, 45	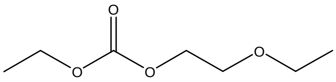	$C_7H_{14}O_4$	162.089
13.3	56, 74, 86, 101, 129, 147	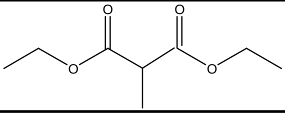	$C_8H_{14}O_4$	174.089
15.7	41, 55, 63, 70 91, 118	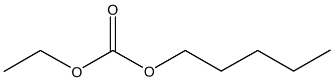	$C_8H_{16}O_3$	160.109
17.7	45, 56, 73, 117, 145	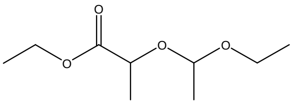	$C_9H_{18}O_4$	190.125
20.4	29, 45, 63, 91	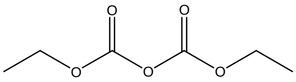	$C_6H_{10}O_5$	162.052
25.5	45, 55, 74, 79, 83, 91, 102, 129	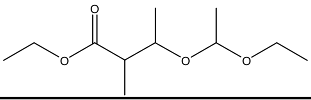	$C_{11}H_{22}O_4$	218.151
26.5	45, 63, 91, 117, 145, 161	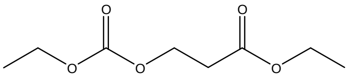	$C_8H_{14}O_5$	190.084

Supplementary Table 5: List of products formed in the liquid phase for irradiated DEC.

The sample was measured by GC-EI/MS and irradiated at a dose of 100 kGy.

Supplementary Note 1: List of abbreviations (alphabetical order)

CLIO - Centre Laser Infrarouge d'Orsay

DEC - Diethyl Carbonate

DFT - Density functional theory

DIMS - Differential Ion Mobility Spectrometry

DMC – Dimethyl Carbonate

EI – Electron impact

EI/MS – Electron impact-Mass spectrometry

ESI – Electrospray ionization

ESI/MS - Electrospray ionization – Mass spectrometry

FEL – Free electron Laser

FT-ICR – Fourier transform ion cyclotron resonance.

GC-EI/MS – Gas chromatography - Electrospray ionization – Mass spectrometry

GC-MS – Gas chromatography - Mass spectrometry

HRMS – High Resolution Mass Spectrometry

IMS – Ion Mobility Spectrometry

IR – Infrared

IRMPD – Infrared Multi Photon Dissociation

LIB – Lithium Ion Batteries

Supplementary Discussion:

High Resolution Mass Spectrometry (HRMS) with an electrospray ionization source to determine the exact mass of formed species upon irradiation.

The most abundant ion is the protonated one ($(DEC+H)^+$) detected at *m/z* 119.079 (Supplementary Figure 3). Two other ions are also detected. One (*m/z* 145.091) corresponds to the $\mathbf{[C_7H_{12}O_3+H]^+}$compound, the other (*m/z* 191.098) to the $\mathbf{[C_8H_{14}O_5+H]^+}$ ion. Interestingly, the latter ion corresponds to a chromatogram peak (t_r = 26.5 min) observed by GC-MS (Supplementary Table 5) and associated with the C_2H_5-O-CO-O-$\mathbf{C_2H_4}$-CO-O-C_2H_5 structure. In addition, the MS spectrum exhibits additional minor peaks at *m/z* values of 147.101, 161.116, 163.096, 175.096 and 219.158 which correspond to $\mathbf{[C_7H_{14}O_3+H]^+}$ (t_r = 3.3 or 6.4 min), $\mathbf{[C_8H_{16}O_3+H]^+}$ (t_r = 15.7 min), $\mathbf{[C_7H_{14}O_4+H]^+}$ (t_r = 11.7 min), $\mathbf{[C_8H_{14}O_4+H]^+}$ (t_r = 13.3 min), and $\mathbf{[C_{11}H_{22}O_4+H]^+}$ (t_r = 25.5 min) ions, respectively, as identified by GC-EI/MS (Supplementary Table 5).

To corroborate the structure of the *m/z* 191.098 ion, a Collision Induced Dissociation (CID) spectrum was also recorded. We have checked elsewhere by Differential Ion Mobility Spectrometry (DIMS) experiment that only one species is present at this *m/z* ratio (see below). The MS/MS spectrum obtained is presented in the Supplementary Figure 4. The peaks detected at *m/z* 145.055, 119.076, 101.065, 91.044 and 73.044 are consistent with the loss of C_2H_5OH, $C_3H_4O_2$, $C_3H_6O_3$, $C_5H_8O_2$ and $C_5H_{10}O_3$, respectively. As shown in Supplementary Figure 5, MS/MS fragmentation patterns of the *m/z* 191.098 ion agree well with the C_2H_5-O-CO-O-C_2H_4-CO-O-C_2H_5 structure already observed by the GC-MS technique (Supplementary Table 5 at t_r = 26.5 min).

DIMS experiments coupled to the MS/MS technique

Differential Ion Mobility Spectrometry (DIMS) enables to separate ions. A set-up integrating a DIMS device to the Esquire MS/MS instrument was used. Details on the set-up can be found elsewhere.[5] DIMS- and mass-selected ions can thus be interrogated by IRMPD spectroscopy. Dispersion voltage was on the order of 1.5 kV, and the compensation voltage (CV) was ramped from -5 to 10 V with a step size of 0.05 V. Mass spectra were averaged from 20 accumulations.

Supplementary Figure 7 displays the DIMS chromatograms of the two main ions resulting from the irradiation of DEC: the *m/z* 145.1 (blue straight line) and 191.1 (red dotted line) ions. In the case of the *m/z* 145.1 ions, one peak at -0.59 V and two partially resolved peaks at 1.56 V and 4.07 V are observed. In the case of the *m/z* 191.1 ion, a single broad mobility peak (the difference between compensation voltages being about 4 V) is detected, with a maximum at about 0.94 V. MS/MS experiments on DIMS- and mass-selected ions can be simply carried out. Using a fixed CV value, ions are selected through the DIMS device and accumulated into the quadrupole ion trap. Ions of interest can be then be mass-selected and subjected to Collision Induced Dissociation (CID).

MS/MS spectra of the *m/z* 145.1 ions selected using two CV values (a) -0.59 V, and b) 4.07 V are given in Supplementary Figure 8. The CID MS/MS spectra are essentially the same when CV is fixed at 1.56 or 4.07 V. The *m/z* 117.1 corresponding to the loss of ethylene is the most abundant fragment, and two weak peaks are detected at *m/z* 73.1 and *m/z* 55.1, corresponding to the loss of $C_3H_4O_2$ and $C_3H_6O_3$, respectively. The MS/MS CID spectrum recorded with the CV fixed at -0.59 V is different since an additional fragment peak can be detected at *m/z* 91.1, which corresponds to the neutral loss of C_4H_6.

A detailed structural analysis based on the DIMS-chromatogram is difficult and out of the scope of this paper. Indeed, mobility under high electric field as applied under DIMS

conditions is not well understood, and no structural information can be directly derived. Based on the DIMS chromatogram, we would rather simply conclude that multiple isomers and/or conformers of the *m/z* 145.1 are likely to be formed. This structural variety is also consistent with the MS/MS CID data.

Supplementary References

1 Gachot, G. et al. Deciphering the multi-step degradation mechnanisms of carbonate-based electrolyte in Li batteries. J. Power Sources 178, 409-421, (2008).

2 Gireaud, L. et al. Mass spectrometry investigations on electrolyte degradation products for the development of nanocomposite electrodes in lithium ion batteries. Anal. Chem. 78, 3688-3698, (2006).

3 Kumai, K. et al. Gas generation mechanism due to electrolyte decomposition in commercial Lithium-ion cell. J. Power Sources 81, 715-719, (1999).

4 Yoshida, H. et al. Degradation mechanism of alkyl carbonate solvents used in Lithium-ion cells during initial charging. J. Power Sources 68, 311-315, (1997).

5 Isenberg, S. L. et al. Optimization of Peptide Separations by Differential Ion Mobility Spectrometry. J. Am. Soc. Mass Spectrom 25, 1592-1599, (2014).
